# Prosocial behaviors as a serial mediator in the association between psychological resilience and meaning in life among sports science students: a cross-sectional study

**DOI:** 10.3389/fpsyg.2026.1770020

**Published:** 2026-02-27

**Authors:** Büşra Ulutürk, Mehmet Behzat Turan, Vesile Şahiner Güler, Hayati Arslan, İbrahim Dalbudak, Berat Koçyiğit, Olcay Mülazımoglu, Melih Balyan, Osman Pepe

**Affiliations:** 1 Faculty of Sports Sciences, Department of Accessible Services, Bitlis Eren University, Bitlis, Türkiye; 2 Faculty of Sports Sciences, Department of Recreation, Erciyes University, Kayseri, Türkiye; 3 Institute of Health Sciences, Physical Education and Sports Sciences, Erciyes University, Kayseri, Türkiye; 4 Faculty of Sports Sciences, Department of Sports Management, Erciyes University, Kayseri, Türkiye; 5 Faculty of Sports Sciences, Department of Sports Management, Uşak University, Uşak, Türkiye; 6 Faculty of Sports Sciences, Department of Sports Management, Süleyman Demirel University, Isparta, Türkiye; 7 Faculty of Sports Sciences, Coaching Education Department, Muğla Sıtkı Koçman University, Muğla, Türkiye; 8 Faculty of Sports Sciences, Physical Education and Sports Department, Ege University, İzmir, Türkiye

**Keywords:** meaning of life, prosociality, psychological resilience, sports science students, sports sciences

## Abstract

**Background:**

This study explored the relationships among psychological resilience, prosocial behaviors, and meaning in life among students in sports science faculties. Meaning in life is a critical factor for individuals preparing for teaching and coaching careers. It provides motivation, purpose, and direction. Psychological resilience supports effective professional functioning by helping individuals cope with challenges. It also enables them to maintain their well-being. Understanding the model that links resilience and meaning in life is crucial. This understanding is important for promoting students’ personal and professional development.

**Methods:**

The study included 1,288 sports science students from 28 universities across Türkiye’s seven geographical regions. Participants were selected through purposive sampling methods. They completed validated scales assessing psychological resilience, prosocial behaviors, and meaning in life. Data were analyzed using SPSS 25 and AMOS 24. Correlation, regression, and mediation analyses were conducted to examine direct and indirect relationships among the variables.

**Results:**

Psychological resilience positively predicted prosocial behaviors. It also positively predicted meaning in life. Prosocial behaviors were found to positively predict a sense of meaning in life. Mediation analysis showed that prosocial behaviors partially mediated the relationship between psychological resilience and meaning in life. This finding suggests that resilient students are more likely to engage in prosocial actions. These actions, in turn, enhance their sense of life purpose.

**Conclusion:**

The findings indicate that prosocial behaviors play a key role in strengthening the link between psychological resilience and meaning in life. The mediation analysis showed that psychological resilience does not directly predict meaning in life; rather, its effect emerges primarily through prosocial behaviors. These results suggest that integrating resilience-building and prosocial behavior–oriented activities into teacher and coach education programs may be an effective strategy to enhance students’ sense of meaning and well-being. Such targeted interventions not only contribute to students’ personal growth but also strengthen their professional readiness, highlighting their practical importance for educational and training contexts.

## 1 Introduction

### 1.1 Conceptual framework of prosocial behaviors

Prosocial behaviors refer to voluntary actions that benefit others and strengthen social harmony (Esmer and Özdaşlı, 2018). Adler describes these actions as expressions of “social interest” (Lauri and Calleja, 2019). Such behaviors help individuals establish meaningful social ties and protect them from social exclusion (Klein, 2017). Engaging in prosocial actions also contributes to one’s personal sense of meaning, aligning with the idea that people continuously pursue purposeful engagement throughout life (Schnell and Hoof, 2012; Hunter et al., 2010). Longitudinal research indicates that multiple dimensions of prosocial behavior positively predict university students’ sense of meaning in life, partly mediated by perceived social support (He et al., 2023).

Beyond social support, prosocial behaviors may influence psychological resilience and meaning in life through several additional mechanisms. Acting prosocially has been shown to enhance self-efficacy, moral identity, and perceived personal competence, which in turn strengthen individuals’ capacity to cope with adversity (Bandura, 1997; Aquino and Reed, 2002). Furthermore, helping behaviors can foster positive affect and cognitive reappraisal, enabling individuals to reinterpret stressful experiences in a more adaptive and meaningful way (Fredrickson, 2001; Park, 2010). These internal psychological gains suggest that prosociality may function not only as a social resource but also as a self-regulatory and motivational process that supports resilience and meaning making.

However, existing research has rarely connected these findings within a broader motivational framework that explicitly incorporates the unique contextual demands of sports sciences education.

### 1.2 Meaning in life and its relationship with prosocial behaviors

Meaning in life is a central human motivation guided by goals that transcend the self (Van Tongeren et al., 2016). Meaning emerges when individuals freely choose responsibilities aligned with their values (Fabry, 1980; Frankl, 2009). As a uniquely human process, meaning making is closely tied to cognitive functioning (Emmons, 2003). Higher levels of meaning also predict later prosocial behaviors, partially through psychological resources such as hope and optimism (Zhang et al., 2022).

Previous studies have typically operationalized meaning in life using two dimensions: the presence of meaning (the extent to which individuals perceive their lives as meaningful) and the search for meaning (the degree to which individuals actively seek meaning) (Steger et al., 2006). While many studies report positive associations between meaning and prosociality, some inconsistencies have emerged, particularly regarding the search for meaning, which has been linked to both adaptive and maladaptive outcomes depending on cultural context and developmental stage (Park, 2010). These mixed findings suggest that the functional role of meaning-related processes may vary across populations, underscoring the need for context-specific investigations such as those conducted within sports sciences education.

Despite this growing evidence, the reciprocal dynamics between meaning in life and prosocial behaviors have not been empirically integrated with psychological resilience in a unified model.

### 1.3 Psychological resilience and the search for meaning

Psychological resilience involves adapting to adversity and maintaining psychological functioning despite challenges (Beasley et al., 2003; Yu and Zhang, 2007). Individuals who can find meaning even under challenging experiences tend to exhibit stronger perseverance (Altıntaş and Gültekin, 2005). Research further indicates that students with greater psychological capital and social support maintain prosocial tendencies during stressful periods, suggesting that prosocial behaviors may serve as a protective factor for resilience (Liu et al., 2025).

Recent findings demonstrate that academic resilience mediates the association between meaning in life and subjective happiness among university students, highlighting resilience as a crucial psychological resource in meaning making processes (Klutsey and Mahama, 2025).

In prior research, psychological resilience has been measured through components such as perseverance, emotional regulation, adaptability, and optimism (Connor and Davidson, 2003). However, many studies conceptualize resilience primarily as an individual trait, often overlooking behavioral expressions such as prosocial engagement that may actively sustain resilience in real-world contexts. This limitation may partially explain why the behavioral pathways linking resilience to meaning in life remain underexplored.

However, evidence regarding how resilience translates into greater meaning in life through behavioral mechanisms such as prosociality remains theoretically suggested but empirically underdeveloped.

### 1.4 Prosocial behaviors within Bonanno’s self-regulation and cognitive control theory

Bonanno’s theory highlights flexibility, social connectedness, and meaningful goals as core principles of resilience (Bonanno, 2004, 2011). The Principle of Flexibility emphasizes regulating emotions across changing contexts, while the Principle of Social Support underscores that giving and receiving support enhances adaptive functioning. The Principle of Meaning and Purpose explains how meaningful goals strengthen coping. These principles collectively suggest that prosocial behaviors may enhance both resilience and life meaning, supporting a possible mediating role (He et al., 2023; Zhang et al., 2022; Liu et al., 2025).

Importantly, although psychological resilience and prosocial behaviors are theoretically related within Bonanno’s framework, they represent conceptually distinct constructs. Psychological resilience refers to an individual’s internal capacity to adaptively regulate emotions, cognitions, and goals in response to adversity, whereas prosocial behaviors reflect observable, outward-oriented actions intended to benefit others (Connor and Davidson, 2003; Eisenberg et al., 2006).

Although Bonanno’s emphasis on social connectedness and behavioral flexibility might suggest conceptual overlap, resilience in this model primarily concerns regulatory flexibility and adaptive capacity, whereas prosocial behaviors involve intentional social engagement and moral action within interpersonal contexts (Bonanno and Burton, 2013; Eisenberg et al., 2010).

Accordingly, in the present study, psychological resilience is conceptualized as an antecedent psychological resource, while prosocial behaviors are treated as a distinct behavioral mechanism through which resilience may be associated with meaning in life, rather than as a component or subdimension of resilience itself.

Additionally, recent research highlights that empathy, moral identity, and sense of security strongly predict prosocial behavior among university students, suggesting that prosocial engagement is intertwined with broader socioemotional regulatory processes relevant to resilience (Peng et al., 2024).

However, applications of Bonanno’s model in the domain of sports sciences education are almost nonexistent, and no empirical studies have examined whether prosociality serves as a behavioral mechanism linking resilience to meaning within this population.

Although the theoretical framework suggests that psychological resilience, prosocial behaviors, and meaning in life may be dynamically related, the present study does not aim to test causal mechanisms. Instead, grounded in Bonanno’s Self-Regulation and Cognitive Control Theory, this research examines whether the observed associations among these variables are consistent with a theoretically informed statistical mediation model using cross-sectional data.

### 1.5 Review of the literature: evidence from teacher candidates and university students

Studies with teacher candidates indicate that teaching is perceived as a meaningful and purposeful endeavor (Marshall, 2009). Psychological resilience among university students varies in relation to socioeconomic factors (Durmuş, 2016), and a sense of purpose in life strongly correlates with the presence of meaning (Bundick, 2011). Perceived social support predicts psychological well-being (Doğru, 2018), while social interest enhances coping abilities (Rareshide and Kern, 1991). Meaning in life also predicts life satisfaction, which is closely related to resilience (Yıkılmaz and Demir-Güdül, 2015).

Recent longitudinal evidence further supports the notion that prosocial behaviors increase meaning in life through perceived social support, thereby reinforcing the importance of social mechanisms in student populations (He et al., 2023).

Nevertheless, much of the existing literature is constrained by cross-sectional designs, limited sample sizes, or culturally homogeneous populations, which restricts the generalizability of findings (Zhang et al., 2022; Liu et al., 2025). Moreover, studies focusing on sports science students remain scarce, despite the fact that this group operates in environments characterized by competition, cooperation, and high performance demands conditions that may uniquely shape the interplay between resilience, prosocial behavior, and meaning in life.

However, despite studies examining psychological resilience, prosocial behaviors, and meaning in life separately, there is an apparent lack of research integrating these three constructs within a unified empirical model among sports science students.

Furthermore, prior research commonly relies on small or homogeneous samples, whereas the present study includes 1,288 students from 28 universities, providing substantially greater generalizability.

Most importantly, the psychological resilience → prosocial behavior → meaning in life pathway has not been systematically tested within sports sciences education, despite students in this field frequently engaging in teamwork, confronting performance-related stress, and experiencing intense social interaction demands.

Given the recent evidence linking prosociality to meaning, well-being, and resilience, a comprehensive model that integrates these constructs is both timely and necessary.

In addition, although mediation models involving psychological resilience, prosocial behaviors, and meaning in life have been suggested conceptually, empirical studies testing these mediation mechanisms remain extremely limited. No existing research has examined whether prosocial behaviors function as a mediator between psychological resilience and meaning in life within university populations, and this gap is particularly pronounced in the field of sports sciences education.

Thus, the present study addresses an important and well-documented gap in the literature.

This study provides the first large-scale empirical test of the mediating role of prosocial behaviors in the relationship between psychological resilience and meaning in life, specifically among students in the sports sciences, a population characterized by high interpersonal engagement, performance pressure, and collaborative learning environments.

Moreover, the sample size (*N* = 1,288 across 28 universities) represents one of the largest cohorts examined in this field, enabling unprecedented statistical power and generalizability.

Together, these features highlight the originality and contribution of the current research.

### 1.6 Research questions

Are psychological resilience, prosocial behaviors, and meaning in life statistically associated, and do prosocial behaviors constitute an indirect association between resilience and meaning in life?

Do prosocial behaviors mediate the relationship between psychological resilience and meaning in life?

### 1.7 Research hypotheses

Grounded in the theoretical framework presented above, the present study proposes an integrated model in which psychological resilience (PR) predicts meaning in life (MIL) both directly and indirectly through prosocial behaviors (PB). Although prior research has examined these constructs separately, the mediating mechanisms linking psychological resilience to meaning in life, particularly through prosocial behavior, remain insufficiently tested, especially in large and diverse samples of sports science students. This study, therefore, addresses a notable gap by empirically testing the pathway:

Psychological Resilience → Prosocial Behaviors → Meaning in Life.

In line with this conceptual model, the following hypotheses were formulated:

*H1*: A significant relationship exists among psychological resilience, prosocial behaviors, and a sense of meaning in life.

*H1a*: Psychological resilience has a positive correlation with meaning in life.

*H1b*: Psychological resilience positively predicts prosocial behaviors.

*H2*: Prosocial behaviors are positively associated with a sense of meaning in life.

*H3*: Prosocial behaviors serve as a mediator between psychological resilience and the pursuit of meaning in life.

*H3a*: Psychological resilience indirectly predicts meaning in life by promoting prosocial behaviors.

*H3b*: The indirect effect (PR → PB → MIL) remains significant when controlling for direct effects.

## 2 Method

### 2.1 Sample size determination

To determine the adequacy of the sample size for detecting the indirect effect in the proposed mediation model, which examines the relationships among psychological resilience, prosocial behaviors, and meaning in life, a Monte Carlo power simulation was conducted. Following recommendations for mediation power analysis (Fritz and MacKinnon, 2007; MacKinnon et al., 2004; Preacher and Hayes, 2008), standardized effect-size scenarios were generated for the a and b paths representing minor (a = b = 0.14), medium (a = b = 0.26), and significant (a = b = 0.39) indirect effects while fixing the direct effect at c′ = 0.10. The direct effect was fixed at a small magnitude (c′ = 0.10) to represent a theoretically and empirically plausible residual association after accounting for the mediator. Previous mediation research indicates that, in psychosocial models, the direct effect often becomes substantially attenuated once key mediating mechanisms such as prosocial behaviors are included in the model (MacKinnon et al., 2007; Hayes, 2018). Accordingly, c′ = 0.10 was selected to reflect a conservative estimate of the remaining direct association between psychological resilience and meaning in life, thereby avoiding overestimation of statistical power in the Monte Carlo simulation. Each condition was simulated using 1,000 replications with normally distributed variables. The significance of the indirect effect was evaluated using the Sobel test at *α* = 0.05. Simulation results showed that medium-sized indirect effects reached approximately 80% power with a sample of about n ≈ 200, whereas detecting minor indirect effects required substantially larger samples (approximately n ≈ 700–750). Significant effects were detectable even with smaller samples (n ≈ 100). Given the theoretical structure of the study and previous empirical findings suggesting medium-level associations among psychological resilience, prosocial behaviors, and meaning in life, the achieved sample size of 1,288 sports science students far exceeded the minimum requirements, providing more than adequate power for detecting indirect effects in the mediation model. Consistent with methodological literature emphasizing the limitations of the Sobel test’s normality assumptions, bootstrap confidence intervals (based on 5,000 resamples) were used in the final mediation analyses to ensure robust inference (Hayes, 2018; Preacher and Hayes, 2008). In the present study, Monte Carlo power simulation was conducted based on the Sobel test, which assumes normality of the sampling distribution of the indirect effect. Although this assumption is commonly adopted in mediation analyses, prior research has shown that the distribution of indirect effects may deviate from normality, particularly in finite samples. Accordingly, the results of the Monte Carlo power analysis should be interpreted with appropriate caution. Future studies may benefit from employing alternative approaches, such as bootstrap-based or Bayesian methods, which do not rely on normality assumptions and may provide more robust estimates of indirect effects.

### 2.2 Participants

The study employed a purposeful sampling method (Creswell, 2009), aiming to include participants who could provide relevant and rich information regarding the research variables. To determine the sample, the faculties of sports sciences in Türkiye were first listed alphabetically and numbered, with each geographical region considered separately. In line with the sampling strategy, four universities were randomly selected from each of Türkiye’s seven geographical regions using a lottery method: the numbers representing the universities were written on pieces of paper and placed in seven separate bags, one for each region. Four numbers were drawn from each bag, and the universities corresponding to the selected numbers were included in the study. Consequently, the study sample comprises third- and fourth-year teaching/coaching students from the physical education teaching and coaching departments of 28 universities for the 2024–2025 academic year. The sample was drawn from seven different geographical regions to capture diversity in sports branches, curriculum differences, and cultural backgrounds, thereby enhancing the generalizability of the findings. Overall, the study included 1,288 volunteer students randomly selected from approximately 9,000 third- and fourth-year students enrolled in sports sciences, teaching, and coaching departments in Türkiye during the 2024–2025 academic year.

### 2.3 Research model

This study employed the relational survey model. This model aims to determine the degree of covariance among two or more variables (Karasar, 2011). The study investigated the relationships between psychological resilience, prosocial behaviors, and meaning in life among students in the sports science field.

Additionally, it aimed to investigate whether prosocial behaviors mediate the relationship between psychological resilience and the experience of meaning in life. In multiple mediation models, the effect of an independent variable on a dependent variable is transmitted through one or more mediators, allowing researchers to examine potential causal pathways linking the predictor to the outcome (Hayes, 2018; Hayes, 2022).

### 2.4 Inclusion criteria of participants

The inclusion criteria for participants were: being a 3rd or 4th-year student in the Faculty of Sports Sciences, teaching and coaching department, and having actively participated in sports for at least 5 years. Exclusion criteria included: studying in a different department, having graduated, studying in a class other than the 3rd or 4th grade, and having been active in sports for less than 5 years.

### 2.5 Data collection

In this study, three scales obtained from the literature, as detailed below, were utilized to investigate the role of positive social behavior in enhancing students’ psychological resilience and sense of meaning in life. The scales were administered face-to-face.

#### 2.5.1 Socio-demographic information form

Based on a review of the literature, the researcher created a seven-question form to collect data on participants’ age, gender, department, class, GPA, place of residence, and income level.

Table 1 includes the distribution of demographic information of the participants.

**Table 1:** Demographic characteristics of the sports science students (*N* = 1,288).

Variables	Groups	*N*	%
Gender	Male	710	55.1
	Female	578	44.9
Age	18–21	569	44.2
	22–25	677	52.6
	26–29	42	3.3
Department	Teaching	607	47.1
	Coaching	681	52.9
Grade	3rd Grade	989	76.8
	4th Grade	299	23.2
Gpa	2.00–2.50	156	12.1
	2.51–3.00	629	48.8
	3.01–3.50	449	34.9
	3.51–4.00	54	4.2
Place of living	Dormitory	180	14.0
	Family house	943	73.2
	Student house	165	12.8
Income level	0–2000	471	36.6
	2001–4,000	316	24.5
	4,001–6,000	168	13.0
	6,001–8,000	137	10.6
	8,001 +	196	15.2

#### 2.5.2 Adult prosociality scale

The scale, first developed by Caprara et al. in 2005, was adapted to Turkish culture by Bağcı and Samur (2016). This scale measures an individual’s positive social behaviors within their community, including sharing, helping, caring, and empathy. It consists of 16 items and uses a 5-point Likert scale. Participants are asked to respond by selecting the option that best reflects their behavior. Higher scores indicate greater prosocial behavior. According to the exploratory factor analysis conducted by Bağcı and Samur (2016) to assess the validity of the adaptation, all items of the scale loaded onto a single dimension (Tables 2–5).

**Table 2:** Adult prosociality scale statistics.

Statistic	Value
Cronbach’s alpha (Mother form)	0.70
Cronbach’s alpha (Father form)	0.91

**Table 3:** Confirmatory factor analysis results for the adult prosociality scale.

Item	Estimate	SE	*Z*	*p*
Item 1	0.65	0.02	30.2	<0.001
Item 2	0.52	0.02	25.7	<0.001
Item 3	0.58	0.02	27.6	<0.001
Item 4	0.63	0.03	23.9	<0.001
Item 5	0.63	0.02	28.8	<0.001
Item 6	0.67	0.02	31.4	<0.001
Item 7	0.70	0.02	32.5	<0.001
Item 8	0.58	0.02	24.8	<0.001
Item 9	0.61	0.02	25.6	<0.001
Item 10	0.61	0.02	25.3	<0.001
Item 11	0.51	0.03	19.3	<0.001
Item 12	0.66	0.02	28.9	<0.001
Item 13	0.65	0.02	30.4	<0.001
Item 14	0.57	0.02	24.1	<0.001
Item 15	0.53	0.02	22.1	<0.001
Item 16	0.52	0.03	22.15	<0.001

**Table 4:** Goodness-of-fit indices for the confirmatory factor analysis of the adult prosociality scale.

X^2^/df	CFI	TLI	RMSEA	*p*
1.96	0.92	0.97	0.01	<0.001

**Table 5:** Meaning in life scale statistics.

Statistic	Value
Cronbach’s alpha (Total)	0.86
Cronbach’s alpha (Presence)	0.87
Cronbach’s alpha (Search)	0.88
Test–retest reliability	*r* = 0.81

#### 2.5.3 Meaning in life scale

Adapted to Turkish culture by Steger et al. (2006) and applied to university students by Demirbaş (2010), the Meaning in life scale consists of 10 items, with all but one (item 9) being positive statements. Item 9, a negative statement, was reverse-scored. The scale comprises two subscales, each representing distinct constructs, and these subscales can be used independently of one another. The maximum total score for each subscale is 35. A high score on the “presence of meaning in life” subscale indicates that the individual perceives their life as meaningful. A high score on the “search for meaning in life” subscale indicates that the individual is actively seeking or exploring meaning in their life. To obtain a total score for the entire scale, the items related to the “search for meaning in life” dimension must be reverse-scored (Steger et al., 2006; Demirbaş, 2010; Tables 6–8).

**Table 6:** Confirmatory factor analysis results for the meaning in life scale.

Dimension	Item	Estimate	SE	*Z*	*p*
Searching for meaning in life	Item 2	0.41	0.05	8.25	<0.001
	Item 3	0.50	0.05	10.60	<0.001
	Item 7	0.58	0.05	12.32	<0.001
	Item 8	0.40	0.05	7.62	<0.001
	Item 10	0.45	0.06	22.00	<0.001
The existence of meaning in life	Item 1	1.05	0.04	27.21	<0.001
	Item 4	1.13	0.04	29.69	<0.001
	Item 5	1.24	0.03	35.90	<0.001
	Item 6	1.00	0.04	25.86	<0.001
	Item 9	0.57	0.06	10.13	<0.001

**Table 7:** Goodness-of-fit indices for the confirmatory factor analysis of the meaning in life scale.

X^2^/df	CFI	TLI	RMSEA	*p*
2.94	0.93	0.97	0.03	<0.001

**Table 8:** Psychological resilience scale for adults statistics.

Statistic	Value
Cronbach’s Alpha (Employee Sample)	0.68–0.79
Cronbach’s Alpha (Student Sample)	0.66–0.81
Cronbach’s Alpha (Total)	0.86

#### 2.5.4 Psychological resilience scale for adults

The scale, developed by Friborg et al. (2003) and adapted for use in Türkiye by Basım and Çetin (2011), consists of a total of 33 questions and employs a 5-point Likert scale. Higher scores on the scale indicate greater psychological resilience. The six-factor scale includes: ‘perception of the future’ and ‘structural style,’ each with four items; ‘family cohesion,’ ‘social competence,’ and ‘self-perception,’ each with six items; and ‘social resources (Basım and Çetin, 2011; Tables 9–11).

**Table 9:** Confirmatory factor analysis results for the psychological resilience scale for adults.

Dimension	Item	Estimate	SE	*Z*	*p*
Perception of self	Item 1	0.42	0.03	8.61	<0.001
	Item 2	0.79	0.04	22.39	<0.001
	Item 3	0.40	0.03	8.80	<0.001
	Item 4	0.84	0.03	24.22	<0.001
	Item 5	0.53	0.04	15.21	<0.001
	Item 6	0.42	0.03	10.42	<0.001
Perception of future	Item 7	0.53	0.04	13.37	<0.001
	Item 8	0.44	0.04	12.23	<0.001
	Item 9	0.42	0.04	10.36	<0.001
	Item 10	0.91	0.04	24.32	<0.001
Structural style	Item 11	0.45	0.04	6.97	<0.001
	Item 12	0.43	0.05	9.39	<0.001
	Item 13	0.41	0.04	2.56	<0.001
	Item 14	0.71	0.05	14.06	<0.001
Social competence	Item 15	0.42	0.04	12.11	<0.001
	Item 16	0.75	0.03	21.62	<0.001
	Item 17	0.41	0.04	8.13	<0.001
	Item 18	0.64	0.04	15.41	<0.001
	Item 19	0.45	0.04	10.02	<0.001
	Item 20	0.79	0.03	22.85	<0.001
Social resources	Item 21	0.50	0.04	7.08	<0.001
	Item 22	0.45	0.04	12.32	<0.001
	Item 23	0.70	0.04	19.21	<0.001
	Item 24	0.44	0.04	12.29	<0.001
	Item 25	0.55	0.04	14.85	<0.001
	Item 26	0.72	0.03	21.33	<0.001
	Item 27	0.40	0.04	11.42	<0.001
Family cohesion	Item 28	0.44	0.04	10.49	<0.001
	Item 29	0.58	0.03	18.89	<0.001
	Item 30	0.66	0.04	18.10	<0.001
	Item 31	0.54	0.03	15.55	<0.001
	Item 32	0.84	0.04	21.52	<0.001
	Item 33	0.75	0.04	20.90	<0.001

**Table 10:** Goodness-of-fit indices for the confirmatory factor analysis of the psychological resilience scale for adults.

X^2^/df	CFI	TLI	RMSEA	*p*
1.15	0.95	0.95	0.04	<0.001

**Table 11:** Descriptive values of the subdimensions of the scales.

Variable	*N*	Min.	Max.	X	SD	Skewness	Kurtosis
Prosocialness	1,288	20.00	75.00	59.09	7.54	−0.38	0.44
Assessing the presence	1,288	5.00	35.00	23.63	6.44	−0.61	0.32
Search for meaning in life	1,288	5.00	35.00	22.97	4.63	−0.29	0.63
Structural style	1,288	7.00	20.00	13.68	2.79	0.22	−0.15
Perception of the future	1,288	4.00	20.00	13.78	3.14	0.14	−0.28
Family cohesion	1,288	10.00	30.00	21.21	3.79	0.00	−0.49
Perception of self	1,288	11.00	30.00	21.67	4.00	0.17	−0.36
Social competence	1,288	10.00	30.00	21.02	4.25	0.21	−0.55
Social resources	1,288	11.00	35.00	25.46	4.74	0.05	−0.56
Psychological resilience	1,288	77.00	161.00	116.83	16.69	0.43	−0.63

### 2.6 Analysis of data

The data used in this research were analyzed using SPSS 25 and SPSS AMOS 24 programs. The dataset was initially examined for erroneous values, outliers, normality, and multicollinearity. Since the kurtosis and skewness values remained within the range of −1 to +1, parametric analyses were performed (Tabachnick and Fidell, 2007). The distribution of the study data was found to be normal. Path analysis using structural equation modeling was employed to test the relationships between the variables. In addition to Pearson correlation analysis, Fisher’s Z transformation test was used to compare the relationships among the variables. Regression analysis was conducted to determine the relationship between participants’ adult prosociality scores and their scores on the Meaning in Life and Psychological Resilience for Adults scales. To ensure the robustness of parameter estimates within the structural equation model, the bootstrap resampling method was applied with 5,000 bootstrap samples. Bias-corrected confidence intervals were used to evaluate the significance and stability of indirect and direct effects. Although mediation analysis was employed using structural equation modeling, the cross-sectional nature of the data precludes causal interpretation. Therefore, the mediation model should be interpreted as a theoretically grounded statistical representation of indirect associations among variables rather than evidence of temporal or causal mechanisms.

## 3 Results

Correlation analyses presented in Table 12 indicated a differentiated pattern of associations among prosocialness, meaning in life, and psychological resilience dimensions. Prosocialness exhibited predominantly weak to low-to-moderate correlations with family cohesion (*r* = 0.08, *Z* = 0.08), presence of meaning in life (*r* = 0.23, *Z* = 0.23), search for meaning in life (*r* = 0.32, *Z* = 0.33), and resilience-related subdimensions such as structural style (*r* = 0.22, *Z* = 0.22), perception of future (*r* = 0.23, *Z* = 0.23), perception of self (*r* = 0.19, *Z* = 0.19), social competence (*r* = 0.21, *Z* = 0.21), and social resources (*r* = 0.16, *Z* = 0.16). These findings suggest that prosocial tendencies are modestly embedded within broader psychosocial resources rather than strongly overlapping with any single construct.

**Table 12:** Correlation coefficients between adult prosociality, meaning in life, and psychological resilience for adult scores (*n* = 1,288).

Dimensions		1	2	3	4	5	6	7	8	9	10
Prosocialness^1^	*r*	1									
Assessing the presence	*r*	0.23^**^	1								
Search for meaning in life^3^	*r*	0.32^**^	0.32^**^	1							
Structural style^4^	*r*	0.22^**^	−0.05	0.13^**^	1						
Perception of future^5^	*r*	0.23^**^	−0.07^*^	0.20^**^	0.44^**^	1					
Family cohesion^6^	*r*	0.08^**^	−0.04	0.01	0.28^**^	0.34^**^	1				
Perception of self^7^	*r*	0.19^**^	−0.07^**^	−0.04	0.40^**^	0.45^**^	0.49^**^	1			
Social competence^8^	*r*	0.21^**^	0.04	0.09^**^	0.34^**^	0.38^**^	0.47^**^	0.50^**^	1		
Social resources^9^	*r*	0.16^**^	0.00	0.01	0.28^**^	0.41^**^	0.57^**^	0.55^**^	0.54^**^	1	
Psychological resilience	*r*	0.24**	−0.04**	0.08**	0.58**	0.66**	0.74**	0.79**	0.76**	0.81**	1

*p < 0.05, **p < 0.001, *N* = 1,288. 1 = Prosocialness; 2 = Assessing the Presence; 3 = Search for Meaning in Life; 4 = Structural Style; 5 = Perception of Future; 6 = Family Cohesion; 7 = Perception of Self; 8 = Social Competence; 9 = Social Resources; 10 = Psychological Resilience.

The presence and search for meaning in life were moderately correlated (*r* = 0.32, *Z* = 0.33), supporting their conceptual relatedness while maintaining empirical distinction. In contrast, associations between meaning in life dimensions and resilience subcomponents were generally weak or negligible, as reflected by low *Z* values (e.g., presence of meaning with structural style: *r* = −0.05, *Z* = 0.05; with family cohesion: *r* = −0.04, *Z* = 0.04), indicating limited direct overlap between meaning-related constructs and resilience resources at the bivariate level. More pronounced associations emerged among psychological resilience subdimensions themselves. Structural style demonstrated moderate to strong correlations with perception of future (*r* = 0.44, *Z* = 0.47), perception of self (*r* = 0.40, *Z* = 0.42), social competence (*r* = 0.34, *Z* = 0.35), and social resources (*r* = 0.28, *Z* = 0.28). Similarly, perception of future showed strong relationships with perception of self (*r* = 0.45, *Z* = 0.48), social competence (*r* = 0.38, *Z* = 0.40), and social resources (*r* = 0.41, *Z* = 0.43). Family cohesion was strongly associated with perception of self (*r* = 0.49, *Z* = 0.53), social competence (*r* = 0.47, *Z* = 0.51), and social resources (*r* = 0.57, *Z* = 0.64), indicating a tightly interconnected resilience structure.

Psychological resilience demonstrated consistently strong correlations with its core subdimensions, particularly perception of future (*r* = 0.66, *Z* = 0.79), family cohesion (*r* = 0.74, *Z* = 0.95), perception of self (*r* = 0.79, *Z* = 1.07), and social competence (*r* = 0.81, *Z* = 1.13). In contrast, its associations with presence (*r* = −0.04, *Z* = 0.04) and search for meaning in life (*r* = 0.08, *Z* = 0.08) were weak, underscoring that resilience and meaning in life represent related yet empirically distinct psychological domains. Overall, Fisher’s *Z* transformations corroborated the observed magnitude of correlation coefficients, with higher *Z* values clustering among resilience-related constructs and lower values characterizing associations involving prosocialness and meaning in life. This pattern suggests that while prosocialness and meaning are meaningfully connected to psychological resilience, their relationships are modest in strength and likely operate through complex, multivariate pathways rather than direct, isolated associations.

Psychological resilience and social competence showed a strong association, suggesting a close potential association. Fisher *Z* scores were reported due to their ability to stabilize the variance of correlation coefficients and facilitate meaningful comparisons across correlations.

The observed relationships among the variables provide preliminary support for a mediation model. Weak to moderate associations between prosocialness and meaning in life, structural style, and social functioning suggest that prosocial behavior may be indirectly influenced by these factors. Stronger associations among structural style, future orientation, family cohesion, self-perception, and social competence indicate potential pathways linking individual and social resources to prosocial tendencies. Additionally, psychological resilience showed strong to strong association with both personal and social attributes, highlighting its potential role as a key mediator within this network.

Multicollinearity diagnostics were examined using tolerance and variance inflation factor (VIF) values. As reported in Table 13, tolerance values for Meaning in Life and Psychological Resilience were 1.00, and the corresponding VIF values were also 1.00. These values are well within acceptable thresholds (tolerance > 0.20; VIF < 5), indicating that multicollinearity was not a concern in the regression analyses and that the parameter estimates can be interpreted with confidence.

**Table 13:** Collinearity diagnostics for regression predictors (tolerance and VIF).

Model	Tolerance	VIF
(Constant)	-	-
Meaning in Life	1.00	1.00
Psychological Resilience	1.00	1.00

Table 14 presents Cook’s Distance values for influential data points in the model. The minimum Cook’s Distance is 0.000, indicating that no observations exert extreme influence on the model. The maximum value is 0.050, suggesting the presence of some potentially influential observations that may affect parameter estimates. The mean Cook’s Distance is 0.001, with a standard deviation of 0.002, indicating that the influence of most data points is minimal and relatively evenly distributed. Although the maximum Cook’s Distance value remains below commonly used critical thresholds, its presence indicates that a small number of observations may exert disproportionate influence on the model. Therefore, additional robustness checks such as re-estimating the model after excluding these cases or conducting sensitivity analyses would help ensure that the reported findings are not driven by a limited number of influential observations and that the results remain stable across different model specifications.

**Table 14:** Cook’s distance values for regression diagnostics.

Min	Max	Mean	Std. deviation
0.000	0.050	0.001	0.002

The regression model summary presented in Table 15 provides an overview of the relationship between resilience, meaning, and prosocial behaviors. The *r* value of 0.40 indicates a moderate positive correlation between the predictor(s) and the outcome variable. The R-squared value of 0.16 means that 16% of the variance in the dependent variable is explained by the independent variable(s). The adjusted R-squared, which accounts for the number of predictors, is also 0.16, suggesting that the inclusion of predictors does not substantially improve the model. Finally, the standard error of the estimate is 6.92, reflecting the average distance between the observed values and the predicted values, indicating a moderate level of variability in the predictions.

**Table 15:** Regression model summary predicting prosocial behavior.

Model	*R*	R square	Adjusted R square	Std. error of the estimate
1	0.40	0.16	0.16	6.92

The ANOVA results presented in Table 16 demonstrate that the regression model is statistically significant. The regression sum of squares (SS = 11,808.66) compared to the residual sum of squares (SS = 61,453.82) indicates that the predictors explain a meaningful proportion of the total variance in the dependent variable. The model yields a highly significant *F*-value [*F*(2, 1,285] = 123.46, *p* < 0.001), confirming that the set of predictor variables collectively makes a statistically significant contribution to explaining variance in the outcome variable. These findings support the overall adequacy and explanatory power of the regression model.

**Table 16:** ANOVA results for the regression model.

Model	Sum of Squares	df	Mean Square	F	Sig.
Regression	11808.66	2	5.904.33	123.46	0.00**
Residual	61453.82	1,285	47.82		
Total	73262.48	1,287			

^**^p < 0.001.

The regression coefficients presented in Table 17 show the contribution of the predictor variables, Meaning in Life and Psychological Resilience, to the dependent variables, including resilience, meaning, and prosocial behaviors. The intercept of the regression model is 34.09, representing the expected value of the dependent variable when all predictors are zero. For Meaning in Life, the unstandardized coefficient (B) is 0.27, indicating that a one-unit increase in Meaning in Life is associated with a 0.27-unit increase in the dependent variable. The standardized coefficient (Beta) is 0.32, reflecting a moderate effect size. The t-value of 12.52 (*p* < 0.001) demonstrates that this predictor is highly significant.

**Table 17:** Regression coefficients predicting prosocial behavior.

Model	*B*	Std. Error	Beta	*t*	*p*
(Constant)	34.09	1.68		20.34	0.00**
Meaning in Life	0.27	0.02	0.32	12.52	0.00**
Psychological Resilience	0.11	0.01	0.24	9.33	0.00**

^**^p < 0.001.

For Psychological Resilience, the unstandardized coefficient (B) is 0.10, meaning that each one-unit increase in Psychological Resilience corresponds to a 0.10-unit increase in the dependent variable. The standardized coefficient (Beta) is 0.24, indicating a smaller effect size compared to Meaning in Life. The t-value of 9.33 (*p* < 0.001) confirms that this predictor is also statistically significant. Overall, both Meaning in Life and Psychological Resilience significantly contribute to the regression model, highlighting their meaningful roles in explaining variations in resilience, meaning, and prosocial behaviors among the participants.

Table 18 shows the appropriate fit index values (Gürbüz, 2021).

**Table 18:** Fit indices for the structural equation model.

Fit criterion	Good fit	Acceptable fit
χ2/df	0 < χ^2^/df < 3	3 < χ^2^/df < 5
RMSEA	0 < RMSEA<0.05	0.05 < RMSEA <0.08
CFI	0.95 < CFI < 1.00	0.90 < CFI < 0.95
TLI	0.95 < TLI < 1.00	0.90 < TLI < 0.95
SRMR	0 < SRMR <0.05	0.05 < SRMR <0.08

The goodness-of-fit indices displayed in Table 19 indicate that the measurement model for adult prosociality, meaning in life, and psychological resilience demonstrates an acceptable to good overall fit. The chi-square value is statistically significant (χ^2^ = 44.59, df = 22), and the chi-square/df ratio of 2.03 falls within the commonly recommended threshold (< 3), indicating an adequate model fit. Incremental fit indices also support the robustness of the model: NFI (0.93), TLI (0.94), and CFI (0.96) all exceed the 0.90 criterion, reflecting strong comparative model performance. Furthermore, the RMSEA value (0.06) remains within acceptable limits (<0.08), indicating a reasonable approximation error. Collectively, these indicators provide evidence that the model fits the data well and supports the structural integrity of the constructs examined.

**Table 19:** Goodness-of-fit values for adult prosociality, meaning in life, and psychological resilience scales.

Model	X^2^	df	X^2^/df	NFI	TLI	CFI	RMSEA
	44.59	22	2.03	0.93	0.94	0.96	0.06

Table 20 indicates a significant relationship between prosocial behaviors and life meaning dimensions as well as psychological resilience (*R* = 0.44, R^2^ = 0.197, *p* < 0.05). The regression analysis shows that the presence of meaning in life (t = 3.08, *p* = 0.002) and the search for meaning (t = 4.17, *p* < 0.001) significantly predict prosocial behaviors, together explaining 17.4% of the variance (*F* = 8.46, *p* < 0.05). Although these associated are statistically significant, the R^2^ values (ranging from 0.16 to 0.197) indicate a modest level of predictive power, suggesting that prosocial behaviors among sport sciences students are influenced by additional psychological, social, and contextual factors not captured in the model. Accordingly, the findings should be interpreted as meaningful but partial contributions rather than comprehensive explanations of prosocial behavior. Moreover, in line with prior methodological considerations, model fit indices such as χ^2^ in the SEM analyses should be interpreted with caution, as χ^2^ statistics are known to be sensitive to large sample sizes and may overestimate model misfit in large samples.

**Table 20:** Regression analysis of participants’ prosocial behaviors, meaning in life, and psychological resilience dimensions for adults.

Dependent variable	Independent variables	ß (Standardized)	ß (Unstandardized)	S. E	T	*p*
Prosociality	The existence of meaning in life	0.18	0.26	0.09	3.08	0.00**
	Searching for meaning in life	0.25	0.50	0.12	4.17	0.00**
	Structural style	0.10	0.33	0.21	1.57	0.12
	Perception of the future	0.04	0.11	0.20	0.57	0.57
	Family cohesion	−0.10	−0.25	0.17	−1.49	0.14
	Self-perception	0.14	0.32	0.17	1.86	0.07
	Social competence	0.09	0.21	0.15	1.35	0.18
	Social resources	0.02	0.05	0.15	0.32	0.75

*R* = 0.44, *R*^2^ = 0.20, Adj. R^2^ = 0.17, *F* = 8.46, *p* = 0.00. **p < 0.001.

Table 21 indicates that the total effect of Psychological Resilience on Meaning in Life was not statistically significant (*β* = 0.007, 95% CI [−0.022, 0.036]). When Prosocial Behaviors were included in the model, the direct effect became statistically significant and negative (β = −0.038, 95% CI [−0.067, −0.008]), while the indirect effect via Prosocial Behaviors was positive and significant (β = 0.045, 95% CI [0.033, 0.058]). Additionally, both the partially mediated effect (β = 0.005, 95% CI [0.004, 0.006]) and the completely mediated effect (β = 0.082, 95% CI [0.060, 0.107]) were statistically significant, with the completely mediated effect showing a larger magnitude. Overall, these findings indicate that the relationship between Psychological Resilience and Meaning in Life operates primarily through Prosocial Behaviors.

**Table 21:** Total, direct, and indirect effects of psychological resilience on meaning in life via prosocial behaviors.

Relationship	Effect type	*β*	SD	*t*	Confidence interval	Conclusion
Psychological resilience → Meaning in life	Total	0.007	0.015	0.471	[−0.022, 0.036]	
	Direct	−0.038	0.015	−2.553	[−0.067, −0.008]	Partial mediation
Psychological resilience → Meaning in life (via prosocial behaviors)	Indirect	0.045	0.007	-	[0.033, 0.058]	Fully mediation
	Partially	0.005	0.001	-	[0.004, 0.006]	
	Completely	0.082	0.012	-	[0.060, 0.107]	

**Figure 1:**
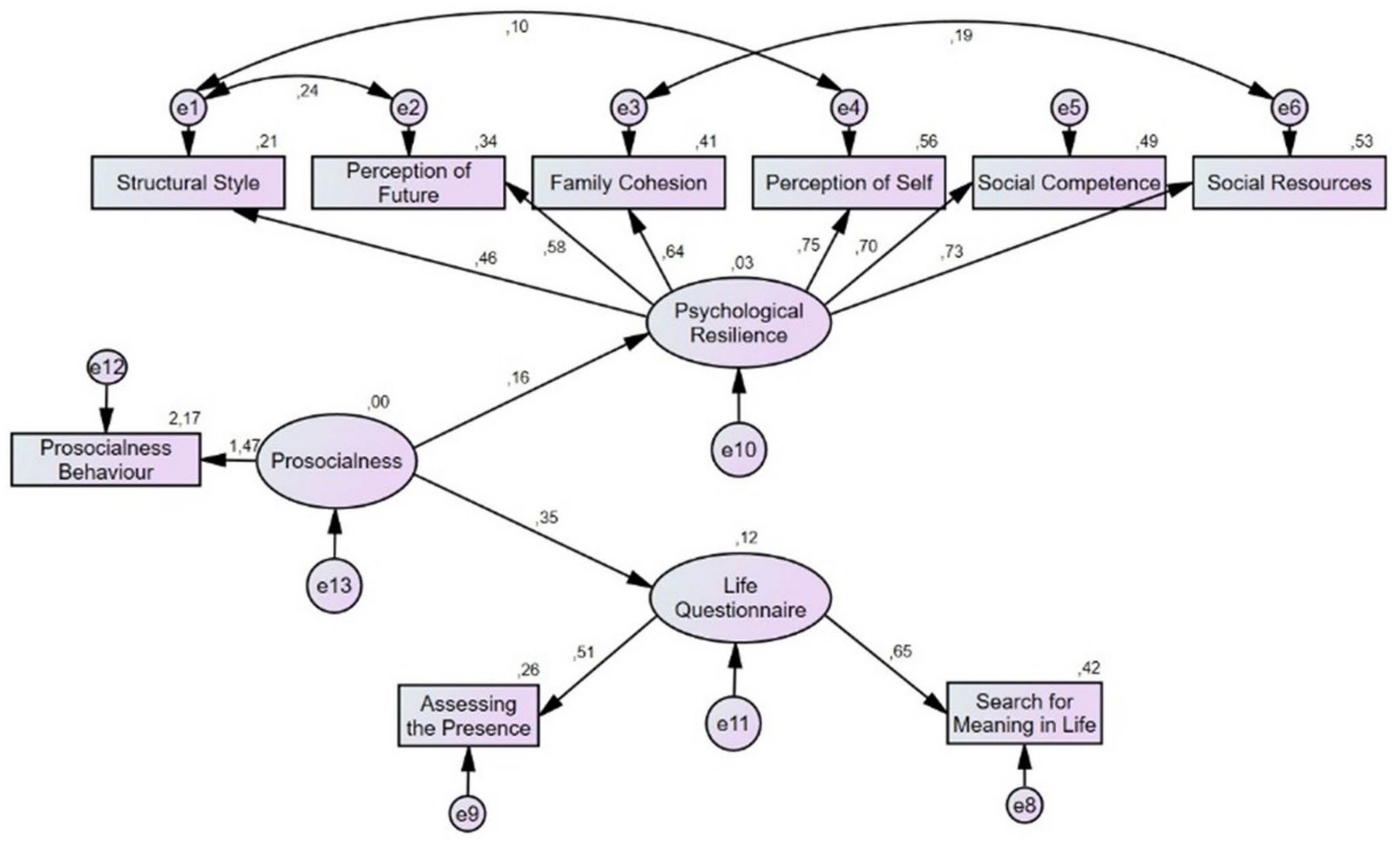
Structural equation model of resilience, prosocial behavior, and meaning in life.

Figure 1 shows the Structural Equation Model of the standard values of the Adult Social Behavior, Meaning in Life, and Adult Resilience Scales.

**Figure 2:**
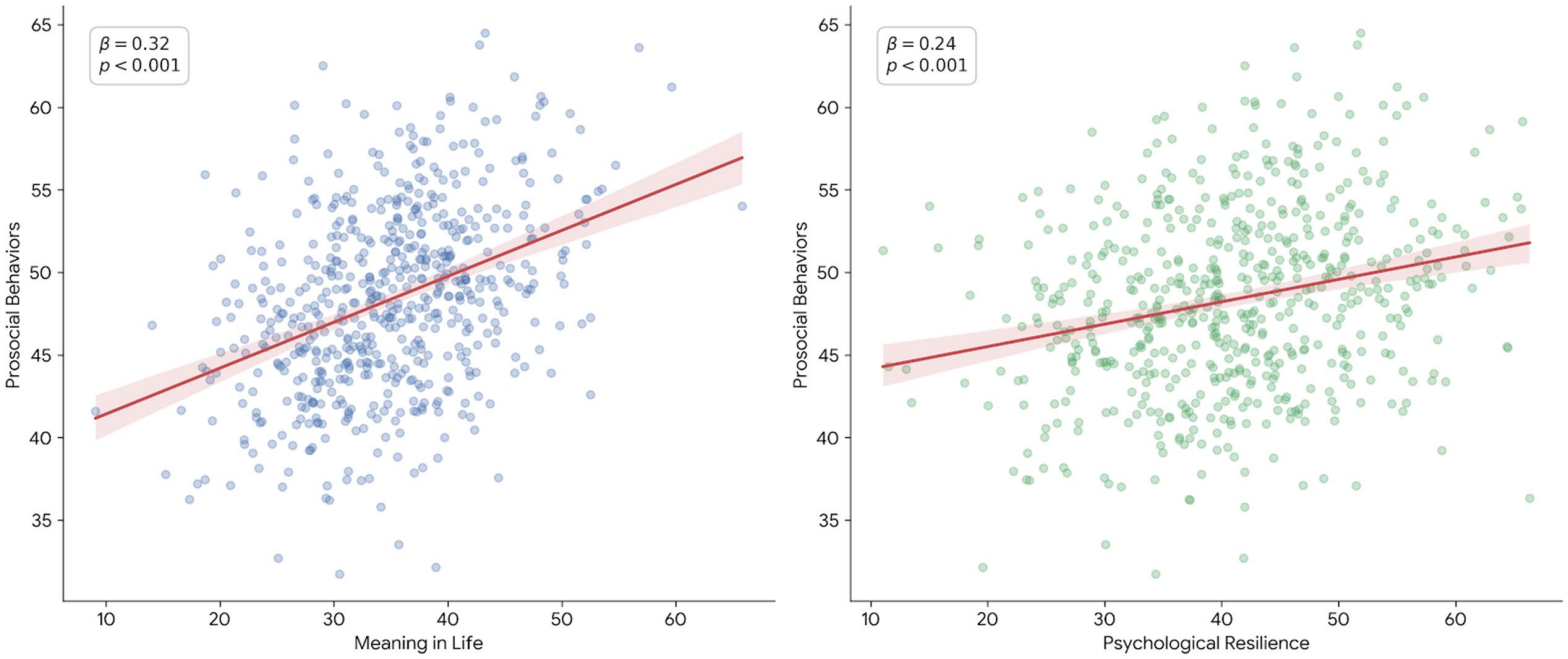
Scatter plots with regression lines showing the relationships between meaning in life, psychological resilience, and prosocial behaviors.

Figure 2 illustrates scatter plots with regression lines depicting the relationships among psychological resilience, prosocial behaviors, and meaning in life. The plots indicate positive linear associations between the variables, such that higher levels of psychological resilience are associated with greater prosocial behaviors, and both constructs are positively related to meaning in life.

## 4 Discussion

This study examined the relationships among psychological resilience, prosocial behavior, and meaning in life among a sample of sports science students, contributing to the growing literature on positive psychological processes in educational and sport-related contexts. The findings indicate that prosocial behavior is positively associated with both the presence of and the search for meaning in life, consistent with prior research reporting links between helping behaviors and indicators of psychological well-being (Kasser et al., 2004; Penner et al., 2005). Similarly, positive associations were observed between resilience-related components such as perception of the future, family cohesion, social competence, and available social resources and prosocial behavior, aligning with studies emphasizing the interconnectedness of social–emotional resources and prosocial tendencies. Beyond these parallels, the present findings are conceptually consistent with recent daily-diary and longitudinal studies reporting bidirectional associations between prosocial behavior and meaning in life (Dakin et al., 2022; Xie et al., 2023; Zhang et al., 2022). Observing comparable relational patterns in sports science students a group characterized by competition, teamwork, and elevated physical and psychological demands extends the relevance of these associations to a distinct academic and performance-oriented population.

Within the context of sports and educational psychology, these findings align with prior evidence suggesting that resilience-related resources and meaning-making processes play a central role in athletes’ adaptive functioning, motivation, and social engagement (Fletcher and Sarkar, 2012; Gucciardi et al., 2008). In educational settings, meaning in life has been associated with greater engagement, persistence, and socially responsible behavior, suggesting that meaning-related processes may operate as motivational resources that support both individual well-being and interpersonal functioning (Hill et al., 2016; Steger, 2012). The present results therefore contribute to an integrative understanding of how resilience and meaning in life jointly relate to prosocial tendencies in sport-oriented educational environments.

The results are also congruent with theoretical perspectives such as Bandura’s Social Cognitive Theory, which emphasizes reciprocal relations among personal factors (e.g., meaning and resilience), behaviors (prosociality), and environmental influences, without implying unidirectional causality (Bandura, 1997). From an Acceptance and Commitment Therapy (ACT) perspective, prosocial actions that align with personal values are theoretically linked to meaning-related processes, a pattern reflected in the observed associations within the proposed model (Hayes, 2022). The primary theoretical contribution of this study lies in testing a comprehensive three-variable structural model within a sports science student sample and demonstrating that prosocial behavior is differentially associated with resilience and the two dimensions of meaning in life. The findings suggest that the presence of meaning and the search for meaning relate to prosocial behavior in distinct ways, indicating that prosociality functions as a differentiated relational mechanism rather than a uniform construct across meaning dimensions. This nuanced pattern adds refinement to the existing literature.

Building on this distinction, the differentiated associations of the presence of meaning and the search for meaning with prosocial behavior provide important insight into the nuanced mechanisms linking psychological resilience, meaning, and social engagement. Specifically, the presence of meaning appears to reflect a more stable and integrated sense of purpose that is readily translated into sustained prosocial engagement, whereas the search for meaning may represent a more exploratory and motivational process that prompts prosocial behavior as a means of identity construction and value clarification (Steger et al., 2006; Park, 2010).

From this perspective, prosocial behaviors may serve distinct psychological functions depending on individuals’ meaning orientation: reinforcing existing meaning structures for those with a strong presence of meaning, while facilitating meaning-making processes for those actively searching for purpose. This distinction helps explain why resilience-related resources may be differentially channeled into social engagement, depending on whether individuals experience meaning as established or emerging (Van Tongeren et al., 2016).

By highlighting these differentiated pathways, the present findings extend existing models of resilience and meaning by demonstrating that prosocial behavior is not merely a uniform outcome of adaptive functioning, but a context-sensitive mechanism through which resilient capacities are socially expressed and meaning-related processes are enacted. This nuanced interpretation strengthens the theoretical contribution of the study by integrating motivational, social, and meaning-based perspectives within a single empirical framework.

Nevertheless, alternative interpretations of the observed relationships should be considered. Prior research suggests that meaning in life may prospectively relate to later prosocial behavior (Zhang et al., 2022), while other studies indicate that engagement in prosocial actions may be associated with subsequent increases in resilience. Accordingly, multiple directional pathways among meaning, prosociality, and resilience remain theoretically plausible. Given the cross-sectional design of the present study, the findings should be interpreted as evidence of associations rather than causal effects, and future longitudinal or experimental research is needed to clarify temporal ordering and directional dynamics. Regarding effect sizes, the regression model explained approximately 16% of the variance in prosocial behavior (R^2^ = 0.16), indicating a statistically significant yet modest level of explained variance. In line with research in psychological and social behavior domains, this finding suggests that meaning in life and psychological resilience are relevant correlates of prosocial behavior, while accounting for only a limited portion of its variability. Prosocial behavior is influenced by a complex interplay of individual, interpersonal, and contextual factors; therefore, a substantial proportion of variance is likely attributable to other unmeasured influences, such as sport identity, team climate, coaching practices, or motivational environments. From this perspective, the present results should be interpreted as reflecting meaningful but partial associations rather than strong predictive effects. Importantly, the acceptable fit indices of the structural model support the adequacy of the model structure, while also underscoring the multifaceted nature of prosocial behavior.

The results of the mediation analysis indicate that psychological resilience does not have a direct influence on meaning in life unless it is expressed through prosocial behaviors. The non-significant total effect suggests that resilience alone may not be sufficient to enhance individuals’ sense of meaning. However, once prosocial behaviors were incorporated into the model, a significant indirect effect emerged, demonstrating that resilience contributes to meaning in life primarily by promoting socially oriented actions such as helping and cooperation.

Notably, the pattern observed in the mediation analysis where the direct effect of psychological resilience on meaning in life becomes negative after the inclusion of prosocial behaviors, while the total effect remains close to zero may be indicative of a statistical suppression effect. Suppression occurs when the inclusion of a mediator increases the predictive validity of an independent variable by accounting for variance that is irrelevant or opposing in direction to the outcome variable (MacKinnon et al., 2000; Paulhus et al., 2004). In the present context, this finding suggests that certain components of psychological resilience may relate to meaning in life in different or even competing ways unless resilience is behaviorally expressed through prosocial engagement.

From a theoretical perspective, this pattern is consistent with models proposing that resilience-related capacities such as emotional control or self-reliance may not inherently foster meaning unless they are translated into socially meaningful action (Bonanno and Burton, 2013; Park, 2010). Prosocial behaviors may therefore function as an activating mechanism that channels resilient capacities toward value-consistent goals, thereby clarifying the positive association between resilience and meaning in life. Similar suppression patterns have been reported in prior mediation research examining complex psychological constructs, where indirect pathways reveal meaningful associations that are obscured at the total-effect level (MacKinnon et al., 2007; Hayes, 2018).

Accordingly, rather than undermining the validity of the mediation model, the observed suppression effect underscores the importance of examining indirect pathways when studying multifaceted constructs such as resilience and meaning. This finding highlights that the contribution of psychological resilience to meaning in life may be conditional upon its expression through prosocial behaviors, offering a more nuanced theoretical interpretation of the mediation process.

One limitation of the present study concerns the assessment of prosocial behavior through self-report measures. Although such instruments are commonly used and effective for capturing individuals’ perceived tendencies, they may be influenced by social desirability, particularly in academic contexts that value ethical conduct, cooperation, and social responsibility. As a result, participants may have overreported prosocial behaviors. In addition to self-report bias, the exclusive focus on sports science students represents a further limitation that may constrain the generalizability of the findings. Students enrolled in sport-related programs may differ from those in other academic disciplines in terms of physical activity levels, team-based experiences, competitive orientation, and exposure to performance-related stressors, all of which may shape resilience, meaning-making processes, and prosocial behavior (Nicholls et al., 2009). Future studies should examine whether similar relational patterns emerge in more diverse student populations and across different educational or athletic contexts. Moreover, although the present study focused on key psychological resources, other potentially relevant variables such as athletic identity, motivational climate, coach–athlete relationships, or organizational culture were not included in the model. Incorporating such contextual factors in future research may provide a more comprehensive understanding of the mechanisms underlying prosocial behavior in sport and educational settings.

In conclusion, this study advances understanding of the relational patterns among meaning in life, psychological resilience, and prosocial behavior in sports science students. The findings highlight meaningful associations among these constructs and suggest that prosocial behavior is closely intertwined with both resilience-related resources and meaning-related processes. These results offer practical implications for educational and sport-based programs aimed at supporting well-being, social functioning, and value-oriented engagement, while underscoring the need for future research designs capable of addressing model.

## 5 Conclusion

The present study provides robust evidence that prosocial behaviors are strongly associated with both meaning in life and psychological resilience among students in sports sciences. By testing these constructs simultaneously within a single structural model, the findings demonstrate that they form a coherent and statistically robust framework in which prosocial behavior occupies a central position rather than representing an isolated outcome. Importantly, the results indicate that psychological resilience does not exert a significant direct effect on meaning in life; instead, its association with meaning in life is largely accounted for when prosocial behaviors are included in the model, underscoring the explanatory role of prosociality within the overall model structure.

The novelty of this study lies in its integrated modeling of prosocial behavior, meaning in life, and psychological resilience constructs that have frequently been examined in isolation or through pairwise associations in prior research. By clarifying how these variables are jointly represented within a single model, the study advances the literature by offering a more comprehensive understanding of their interrelationships in sports science students. From an applied perspective, the findings highlight the practical relevance of incorporating prosocial behavior–oriented components alongside resilience-building efforts in teacher and coach education programs, as such integrated approaches may contribute to students’ sense of meaning, psychological well-being, and professional readiness in educational and training contexts.

## 6 Limitations and future research

- The study was conducted in 28 universities across seven geographical regions.
- Only four universities were selected from each region.
- Increasing the number of universities per region in future studies may produce results that more accurately represent each region.
- Expanding the number of universities would also increase the overall sample size.
- A larger sample size would improve the generalizability of future findings.
- The study included only universities located within Türkiye’s seven geographical regions.
- Future research could include coaching and teaching departments in different countries.
- Including international samples would allow comparative evaluations across educational systems.
- Such comparisons could reveal how different curricula influence prosociality, meaning in life, and psychological resilience across countries.
- The research sample consisted only of students from coaching and teaching departments.
- Future studies could include additional departments within the Faculty of Sports Sciences.
- A limitation of this study is that participation was restricted to students with a minimum of 5 years of active sports participation, which may limit the generalizability of the findings to sports science students with shorter or no formal sport experience.
- Including sports management departments could provide insights into future managerial roles in coaching fields.
- Including recreation departments may offer perspectives on students who work with younger populations.
- Expanding to all sports science departments would enable stronger interdepartmental comparisons.
- The study identified a relationship between prosocial behaviors and the experience of meaning in life.
- Future research could measure students’ scale scores before participation in prosocial activities.
- Future studies could also measure the same students after they have participated in prosocial activities.
- Comparing pre- and post-scores would help determine whether engaging in prosocial actions leads to measurable changes in meaning in life or an increase in prosociality.
- An important limitation of this study is its cross-sectional design, which restricts causal interpretation. Although a mediation model was tested using structural equation modeling, the temporal ordering of psychological resilience, prosocial behaviors, and meaning in life cannot be empirically established. Therefore, the findings should be interpreted as reflecting statistically significant associative and indirect relationships that are consistent with the proposed theoretical framework, rather than as evidence of model. Future longitudinal or experimental studies are needed to examine the directionality and causal processes underlying the relationships among psychological resilience, prosocial behaviors, and meaning in life.

## 7 Educational policy implications

This study contributes to the literature on the mediating role of prosocial behaviors in the relationship between psychological resilience and meaning in life, drawing on Bonanno’s Self-Regulation and Cognitive Control Theory (Bonanno, 2004, 2011). The study’s results suggest that creating opportunities for future teachers and coaches to engage in prosocial behaviors can enhance their psychological resilience and contribute to a greater sense of meaning in their lives (Bonanno, 2004; Bonanno, 2011). Enhancing individuals’ psychological resilience and sense of meaning in life can positively impact their academic success, thereby improving the educational process and their professional preparation.

In line with the study’s purpose, subject, and findings, it is recommended that applied courses be incorporated into the teaching and coaching department curricula, allowing students to engage in prosocial behaviors and participate in activities that promote social solidarity and cooperation. This would better prepare students for their professional roles and foster their development as positive role models.

Courses incorporating in-class group work with shared goals could be added to the curricula of coaching, physical education, and sports teaching students.

Integrating topics and courses related to prosociality and solidarity, including spiritual, cultural, religious, and national examples, into the curricula of teacher and coach candidates could help students develop into better role models in these dimensions.

Universities could establish partnerships with non-governmental organizations to facilitate students’ voluntary participation in these organizations during their education. This would contribute to the development of social responsibility awareness among students in these departments.

## 8 Practical implications

Based on the results of our study, which established a relationship between prosociality and meaning in life, as well as between prosociality and psychological resilience, environments promoting social solidarity and cooperation can be created for physical education and sports teacher and coach candidates. This would allow students to engage in prosocial behaviors, thereby contributing to their development as professional role models and enhancing their psychological resilience against the challenges they may encounter in their careers.

Our study also found that individuals exhibiting more prosocial behaviors are more likely to cope with stress, solve problems, develop diverse perspectives, and find life more meaningful compared to those exhibiting less prosocial and less social behaviors. Fostering a sense of meaning in life, which is crucial for individuals, can positively impact their lives by encouraging them to help others and build social relationships, while also developing their resilience, thus creating a positive cycle.

The research indicates that students in teaching and coaching departments can contribute to the development of students’ prosocial tendencies by incorporating teamwork-based studies and group work into their courses.

## Data Availability

The original contributions presented in the study are included in the article/supplementary material, further inquiries can be directed to the corresponding authors.
